# Machine Learning–Based Prediction of Clinical Outcomes for Children During Emergency Department Triage

**DOI:** 10.1001/jamanetworkopen.2018.6937

**Published:** 2019-01-11

**Authors:** Tadahiro Goto, Carlos A. Camargo, Mohammad Kamal Faridi, Robert J. Freishtat, Kohei Hasegawa

**Affiliations:** 1 Department of Emergency Medicine, Massachusetts General Hospital, Harvard Medical School, Boston; 2 Division of Emergency Medicine, Children's National Health System, Washington, DC; 3 Department of Pediatrics, George Washington University School of Medicine and Health Sciences, Washington, DC; 4 Department of Genomics and Precision Medicine, George Washington University School of Medicine and Health Sciences, Washington, DC

## Abstract

**Importance:**

While machine learning approaches may enhance prediction ability, little is known about their utility in emergency department (ED) triage.

**Objectives:**

To examine the performance of machine learning approaches to predict clinical outcomes and disposition in children in the ED and to compare their performance with conventional triage approaches.

**Design, Setting, and Participants:**

Prognostic study of ED data from the National Hospital Ambulatory Medical Care Survey from January 1, 2007, through December 31, 2015. A nationally representative sample of 52 037 children aged 18 years or younger who presented to the ED were included. Data analysis was performed in August 2018.

**Main Outcomes and Measures:**

The outcomes were critical care (admission to an intensive care unit and/or in-hospital death) and hospitalization (direct hospital admission or transfer). In the training set (70% random sample), using routinely available triage data as predictors (eg, demographic characteristics and vital signs), we derived 4 machine learning–based models: lasso regression, random forest, gradient-boosted decision tree, and deep neural network. In the test set (the remaining 30% of the sample), we measured the models’ prediction performance by computing C statistics, prospective prediction results, and decision curves. These machine learning models were built for each outcome and compared with the reference model using the conventional triage classification information.

**Results:**

Of 52 037 eligible ED visits by children (median [interquartile range] age, 6 [2-14] years; 24 929 [48.0%] female), 163 (0.3%) had the critical care outcome and 2352 (4.5%) had the hospitalization outcome. For the critical care prediction, all machine learning approaches had higher discriminative ability compared with the reference model, although the difference was not statistically significant (eg, C statistics of 0.85 [95% CI, 0.78-0.92] for the deep neural network vs 0.78 [95% CI, 0.71-0.85] for the reference; *P* = .16), and lower number of undertriaged critically ill children in the conventional triage levels 3 to 5 (urgent to nonurgent). For the hospitalization prediction, all machine learning approaches had significantly higher discrimination ability (eg, C statistic, 0.80 [95% CI, 0.78-0.81] for the deep neural network vs 0.73 [95% CI, 0.71-0.75] for the reference; *P* < .001) and fewer overtriaged children who did not require inpatient management in the conventional triage levels 1 to 3 (immediate to urgent). The decision curve analysis demonstrated a greater net benefit of machine learning models over ranges of clinical thresholds.

**Conclusions and Relevance:**

Machine learning–based triage had better discrimination ability to predict clinical outcomes and disposition, with reduction in undertriaging critically ill children and overtriaging children who are less ill.

## Key Points

**Question:**

Do machine learning approaches improve the ability to predict clinical outcomes and disposition of children at emergency department triage?

**Findings:**

In this prognostic study of a nationally representative sample of 52 037 emergency department visits by children, machine learning–based triage models had better discrimination ability for clinical outcomes and disposition compared with the conventional triage approaches, with a higher sensitivity for the critical care outcome and higher specificity for the hospitalization outcome.

**Meaning:**

Machine learning may improve the prediction ability of triage approaches and could be used to reduce undertriage of critically ill children and to improve resource allocation in emergency departments.

## Teaser

This prognostic study uses data from the National Hospital Ambulatory Medical Care Survey to test the ability of 4 machine learning approaches to predict clinical outcomes of children presenting to emergency department triage.

## Introduction

Of 137 million annual emergency department (ED) visits in the United States, 30 million visits are made by children.^1,2,3^ With the steady increase in the volume and acuity of patient visits to EDs,^4^ accurate differentiation and prioritization of patients at the ED triage is important. However, current triage systems have suboptimal ability to differentiate critically ill children,^5,6,7^ and the proportion of children seen by a physician within the time recommended by triage has been declining because of pervasive ED crowding.^8^ Therefore, it is essential to optimize triage systems to not only avoid undertriaging critically ill children but also reduce overtriaging in order to provide high-quality and timely care and to achieve efficient resource allocation in the ED.

Machine learning approaches have attracted attention because of their superior ability to predict patient outcomes compared with traditional approaches in various settings and disease conditions (eg, sepsis and unplanned transfers to the intensive care unit [ICU]).^9,10,11,12,13,14,15^ The advantages of machine learning approaches include their ability to process complex nonlinear relationships between predictors and yield more stable predictions.^16^ For example, a recent 2-center retrospective study using one of the machine learning approaches reported an improved triage classification in a general ED population.^5^ While these prior studies suggest that machine learning approaches may improve the decision-making ability at the ED triage, no study, to our knowledge, has investigated the utility of machine learning approaches to predict clinical outcomes and disposition of children in the ED. Additionally, in the current triage settings with limited resources and time pressure, it is not feasible for ED providers to use all information available without the use of automated machine learning approaches.

To address this knowledge gap, we analyzed nationally representative ED visit data to develop machine learning–based triage models that predict the clinical course of children after ED triage. We also compared their prediction performance to that of the reference model using 5-level conventional triage classification.

## Methods

### Study Design and Setting

This is a prognostic study of combined data from the ED component of the National Hospital Ambulatory Medical Care Survey (NHAMCS) from January 1, 2007, through December 31, 2015.^17^ In brief, NHAMCS is a nationally representative sample of visits to noninstitutional general and short-stay hospitals, excluding federal, military, and Veterans Affairs hospitals, in the 50 US states and the District of Columbia. The survey is conducted annually by the Centers for Disease Control and Prevention (CDC) National Center for Health Statistics. For example, in 2015, the NHAMCS recorded 21 061 representative ED visits from 267 EDs, resulting in a weighted national sample of 137 million ED patient visits. A detailed description of NHAMCS procedures is available in the technical notes section of NHAMCS ED Survey.^17^ The NHAMCS data are publicly available and are provided by the CDC. This study followed the Transparent Reporting of a Multivariable Prediction Model for Individual Prognosis or Diagnosis (TRIPOD) reporting guideline for prognostic studies.^18^ The institutional review board of Massachusetts General Hospital waived review of the current analysis.

### Study Samples

We identified all ED visits made by children (aged ≤18 years). We excluded visits that did not have information on triage classification level at the ED visit, were dead on ED arrival, left before being seen or against medical advice, or had data inconsistencies (ie, systolic blood pressure >300 mm Hg, diastolic blood pressure >200 mm Hg, pulse rate >300/min, respiratory rate >80/min, or oxygen saturation >100%). We focused on the 2007 to 2015 data based on the availability of vital sign information during these years.

### Predictors

The predictors for machine learning models were chosen from routinely available data at ED triage using a priori knowledge.^5,19^ Specifically, the predictors included patient age, sex, mode of arrival (walk-in vs ambulance), vital signs (temperature, pulse rate, systolic and diastolic blood pressure, respiratory rate, and oxygen saturation), visit reasons, patient’s residence (home vs other [eg, long-term care facility]), ED visit in the preceding 72 hours, and patient comorbidities. Visit reasons were grouped based on the reason for visit classification for ambulatory care provided by the CDC.^20^ Patient comorbidities were classified into 12 categories according to the pediatric complex chronic conditions, which include neuromuscular, cardiovascular, respiratory, renal, gastrointestinal, hematologic, immunologic, metabolic, other congenital or genetic defect, malignancy, and premature and neonatal comorbidities.^21,22,23^

### Outcomes

The primary outcome was critical care according to the previous studies.^5,13,19,24^ Critical care—as an indicator for high-severity medical need—was defined as either direct admission to an ICU or in-hospital death.^5,13,19,24^ Timely ED management for patients who require admission to the ICU has been consistently related to improvement in patient outcomes.^25,26,27^ The secondary outcome was hospitalization, defined as either admission to an inpatient care site or direct transfer to an acute care hospital.^5,13,19^

### Statistical Analysis

In the training set (70% random sample), we developed the reference and 4 machine learning models to predict the probability of 2 outcomes. First, as the reference model, we fit a logistic regression model including only the conventional triage classification data recorded in the database.^19^ The NHAMCS data encoded triage as immediate (level 1), emergent (level 2), urgent (level 3), semiurgent (level 4), and nonurgent (level 5). While most EDs used 5-level triage systems (eg, pediatric emergency severity index), 7% of the NHAMCS EDs used other systems that were systematically recoded to the 5-level system by the CDC.^17^ Next, using the predictors above, we constructed 4 machine learning prediction models: (1) logistic regression with lasso regularization (lasso regression),^28^ (2) random forest,^29^ (3) gradient-boosted decision tree,^30^ and (4) deep neural network.^31^ Lasso regularization extends standard regression models by enabling us to select important predictors (feature selection), which is more interpretable and clinically useful (rather than a standard logistic regression model using many predictors). For the lasso regression, we chose the regularization parameter (lambda) that gives the minimal misclassification error rate in order to penalize large coefficients that come from small sample sizes. The minimal lambda was calculated using 10-fold cross-validation using the glmnet package. Random forest is an ensemble of decision trees created by using bootstrap samples of the training data and random feature selection in tree induction. Gradient-boosted decision tree is another ensemble approach—an additive model of decision trees estimated by gradient descent. For the random forest and gradient-boosted tree models, we have used a grid search strategy to identify the best combination of hyperparameters by using the ranger and caret packages.^32^ Deep neural network is a class of machine learning algorithm consisting of multiple layers of nonlinear processing units to learn the value of the parameters that result in the best prediction of outcome. In the deep neural network, we constructed 5-layer feedforward model with adaptive moment estimation optimizer^33^ using Keras implemented in R statistical software version 3.4.2 (RStudio).^31^ For the deep neural network, we developed the final models by randomly and manually tuning the hyperparameters, such as the number of layers and hidden units, learning rate, learning rate decay, dropout rate, batch size, and epochs, using the keras package. To minimize potential overfitting in the 4 machine learning models, we used lasso regularization, cross-validation, out-of-bag estimation, and dropout; ridge regularization; and batch normalization when developing models. In the logistic regression with lasso penalization (regularization), the penalty function shrinks large coefficients toward 0, thereby minimizing potential overfitting.^34^ In the lasso regression and gradient-boosted tree models, we used 10-fold cross-validation to measure the prediction error with a smaller variance than that from a single train-test set split.^35^ Similarly, in the random forest models, we used out-of-bag (left-out samples after bagging) estimation to measure the prediction errors.^36^ In the deep neural networks, to minimize potential overfitting, we used dropout that randomly removes portions of units in the network,^37^ ridge regularization that shrinks large coefficients,^38^ and batch normalization that normalizes the means and variances of layer inputs.^39^

For the predictors with missing data (eTable 1 in the Supplement), we conducted multiple imputation by using the random forest method.^40^ Random forest imputation is a nonparametric algorithm that can accommodate nonlinearities and interactions and does not require a particular parametric model to be specified.^41^ In summary, using this approach, the single point estimates were generated by random draws from independent normal distributions centered on conditional means predicted using random forest. Random forest uses bootstrap aggregation of multiple regression trees to reduce the risk of overfitting, and it combines the estimates from many trees.^40^ Missingness was imputed using all predictors, outcomes, and other covariates (race/ethnicity, ED disposition [eg, discharge against medical advice], and calendar year).

In the test set (30% random sample), we measured the prediction performance of each model by computing (1) C statistics (ie, the area under the receiver operating characteristic [ROC] curve), (2) prospective prediction results (ie, sensitivity, specificity, positive predictive value, negative predictive value, positive likelihood ratio, and negative likelihood ratio), and (3) decision curve analysis. To address the class imbalance in the critical care outcome (ie, the low proportion of outcome), we chose the threshold of prospective prediction results based on the ROC curve (ie, the value with the shortest distance to the perfect model).^16^ The decision curve analysis is a measure that takes into account the different weights of different misclassification types with a direct clinical interpretation (eg, trade-offs between undertriage and overtriage for each model).^42,43^ Specifically, the relative impact of false-negative (undertriage) and false-positive (overtriage) results given a threshold probability (or clinical preference) was accounted to yield net benefit in each model. The net benefit of each model over a specified range of threshold probabilities of outcome was graphically displayed as a decision curve. We have deposited the analysis code to a public repository (https://github.com/HasegawaLab/ED_triage_ML_children).

To gain insights into the contribution of each predictor to machine learning models, we also computed the variable importance in the gradient-boosted decision tree and random forest models for each outcome. The variable importance is a scaled measure to have a maximum value of 100.^32,44^ A DeLong test was used to compare ROC curves.^45^ We considered 2-sided *P* < .05 to be statistically significant. All analyses were performed with R statistical software version 3.4.1 (R Foundation for Statistical Computing).

## Results

Between January 1, 2007, and December 31, 2015, the database recorded 64 042 ED visits by children. Of these, we excluded 10 792 visits without the information on triage classification, 2 deaths on arrival, 1138 who left before being seen or against medical advice, and 73 records with data inconsistencies. We included the remaining 52 037 ED visits in the current analysis. The ED visit characteristics were comparable between the analytic and nonanalytic cohorts (eTable 2 in the Supplement). Of 52 037 visits in the analytic cohort, the median (interquartile range) age was 6 (2-14) years and 24 929 patients (48.0%) were female (Table 1).

**Table 1.** Predictor Variables and Outcomes in 52 037 Children Presenting to the ED

Variable	Value
Age, median (IQR), y	6 (2-14)
Female, No. (%)	24 929 (48.0)
Arrived by ambulance, No. (%)	3637 (7)
Vital signs, median (IQR)	
Temperature, °F	98.4 (97.9-99.2)
Pulse rate, beats/min	105 (87-128)
Systolic blood pressure, mm Hg	109 (104-120)
Diastolic blood pressure, mm Hg	62 (66-72)
Respiratory rate, breaths/min	20 (18-24)
Oxygen saturation, %	99 (98-100)
Reason for visit, No. (%)	
General, eg, fever	9161 (18)
Respiratory related	8201 (16)
Gastrointestinal related	6677 (13)
Musculoskeletal related	4489 (9)
Eye and ear related	2912 (6)
Injuries	10 594 (20)
ED visit from patient’s home, No. (%)	51 584 (99)
ED revisit within 72 h, No. (%)	1390 (3)
≥1 Pediatric complex chronic condition, No. (%)	291 (0.6)
Outcomes, No. (%)	
Critical care outcome^a^	163 (0.3)
Hospitalization outcome^b^	2352 (4.5)

Abbreviations: ED, emergency department; IQR, interquartile range.

^a^ Direct admission to intensive care unit or in-hospital death.

^b^ Admission to an inpatient care site or direct transfer to an acute care hospital.

### Prediction of Critical Care Outcome

**Figure 1.**
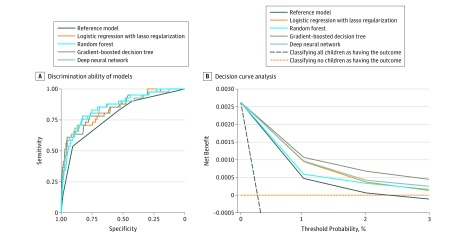
Prediction Ability of the Reference and Machine Learning Models for Intensive Care Unit Use and In-Hospital Mortality in the Test Set A, Receiver operating characteristic curves. The corresponding values of the area under the curve for each model (ie, C statistics) are presented in Table 2. B, Decision curve analysis. The x-axis indicates the threshold probability for critical care outcome. The y-axis indicates the net benefit. The decision curves indicate the net benefit of models (the reference model and 4 machine learning models) as well as 2 clinical alternatives (classifying no children as having the outcome vs classifying all children as having the outcome) over a specified range of threshold probabilities of outcome. Compared with the reference model, the net benefit for all machine learning models was greater over the range of threshold probabilities.

Overall, 163 children (0.3% of 52 037 visits) had a critical care outcome. The discrimination ability of different models, as represented by ROC curves, is shown in Figure 1A.

The reference model had the lowest discriminative ability (C statistic, 0.78; 95% CI, 0.71-0.85) (Table 2), while all 4 machine learning models had a high discriminative ability. For example, the random forest model and deep neural network had nonsignificantly higher C statistics (random forest: 0.85; 95% CI, 0.79-0.91; *P* = .07 and deep neural network: 0.85; 95% CI, 0.78-0.92; *P* = .16). Additionally, compared with the reference model, all machine learning models had a higher sensitivity (eg, 0.54 [95% CI, 0.39-0.69] in the reference model vs 0.78 [95% CI, 0.63-0.90] in the deep neural network) to predict the critical care outcome. By contrast, the reference model had a higher specificity (0.91; 95% CI, 0.75-0.93) compared with the machine learning models (eg, 0.86 [95% CI, 0.69-0.96] for lasso regression). With the low prevalence of critical care outcomes (0.3%), the positive predictive values of all models were low (0.01 [95% CI, 0.01-0.02] in all models) and the negative predictive values were high (0.99 [95% CI, 0.99-0.99] in all models). While the reference model had the highest positive likelihood ratio (5.72; 95% CI, 4.29-7.64), the deep neural network had the lowest negative likelihood ratio (0.26; 95% CI, 0.15-0.47). Specifically, while the reference model identified all critically ill children in triage levels 1 and 2 (immediate and emergent) (53.7% of all critically ill children) (eTable 3 in the Supplement), it failed to correctly identify the remaining critically ill children in triage levels 3 to 5 (46.4% of all critically ill children). By contrast, while the machine learning models failed to identify few critically ill children in triage levels 1 and 2, they correctly identified 47.3% to 68.4% of critically ill children in triage levels 3 to 5. In the decision curve analysis (Figure 1B), compared with the reference model, the net benefit for all machine learning models was greater over the range of threshold probabilities, with gradient-boosted decision tree and deep neural network having the greatest net benefit.

**Table 2.** Prediction Ability of the Reference Model and 4 Machine Learning Models in Children Presenting to the Emergency Department

Outcome and Model	C Statistic (95% CI)	*P* Value^a^	Sensitivity (95% CI)	Specificity (95% CI)	PPV (95% CI)	NPV (95% CI)	PLR (95% CI)	NLR (95% CI)
**Critical Care Outcome**								
Reference model	0.78 (0.71-0.85)	[Reference]	0.54 (0.39-0.69)	0.91 (0.75-0.93)	0.01 (0.01-0.02)	0.99 (0.99-0.99)	5.72 (4.29-7.64)	0.51 (0.37-0.71)
Logistic regression with lasso regularization	0.84 (0.77-0.91)	.29	0.71 (0.56-0.83)	0.86 (0.69-0.96)	0.01 (0.01-0.02)	0.99 (0.99-0.99)	4.96 (4.01-6.07)	0.34 (0.21-0.54)
Random forest	0.85 (0.79-0.91)	.07	0.76 (0.61-0.88)	0.84 (0.70-0.92)	0.01 (0.01-0.02)	0.99 (0.99-0.99)	4.59 (3.84-5.48)	0.29 (0.17-0.50)
Gradient-boosted decision tree	0.84 (0.79-0.92)	.08	0.78 (0.63-0.90)	0.77 (0.52-0.90)	0.01 (0.01-0.02)	0.99 (0.99-0.99)	4.73 (3.40-4.73)	0.27 (0.15-0.49)
Deep neural network	0.85 (0.78-0.92)	.16	0.78 (0.63-0.90)	0.83 (0.62-0.92)	0.01 (0.01-0.02)	0.99 (0.99-0.99)	4.62 (3.92-5.48)	0.26 (0.15-0.47)
**Hospitalization Outcome**								
Reference model	0.73 (0.71-0.75)	[Reference]	0.83 (0.80-0.86)	0.55 (0.52-0.58)	0.08 (0.07-0.08)	0.99 (0.99-0.99)	1.82 (1.75-1.89)	0.33 (0.28-0.38)
Logistic regression with lasso regularization	0.78 (0.76-0.80)	<.001	0.67 (0.64-0.71)	0.75 (0.70-0.78)	0.11 (0.10-0.12)	0.98 (0.98-0.98)	2.71 (2.56-2.88)	0.43 (0.39-0.48)
Random forest	0.80 (0.78-0.81)	<.001	0.74 (0.71-0.77)	0.71 (0.68-0.74)	0.11 (0.10-0.11)	0.98 (0.98-0.99)	2.42 (2.31-2.53)	0.32 (0.28-0.37)
Gradient-boosted decision tree	0.80 (0.78-0.81)	<.001	0.74 (0.71-0.78)	0.71 (0.68-0.74)	0.11 (0.10-0.11)	0.98 (0.98-0.99)	2.41 (2.30-2.52)	0.32 (0.28-0.37)
Deep neural network	0.80 (0.78-0.81)	<.001	0.71 (0.67-0.74)	0.74 (0.71-0.77)	0.11 (0.10-0.11)	0.99 (0.98-0.99)	2.46 (2.31-2.58)	0.35 (0.30-0.40)

Abbreviations: NLR, negative likelihood ratio; NPV, negative predictive value; PLR, positive likelihood ratio; PPV, positive predictive value.

^a^ Comparison of area under the curve of the reference model (conventional triage approaches) with those of each machine learning approach by using a DeLong test.

### Prediction of Hospitalization Outcome

**Figure 2.**
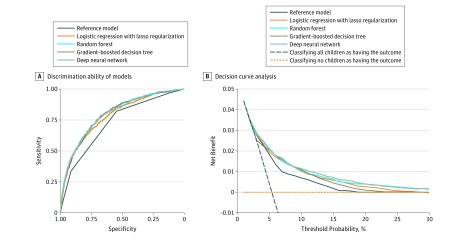
Prediction Ability of the Reference and Machine Learning Models for Hospitalization in the Test Set A, Receiver operating characteristic curves. The corresponding values of the area under the curve for each model (ie, C statistics) are presented in Table 2. B, Decision curve analysis. The x-axis indicates the threshold probability for hospitalization outcome. The y-axis indicates the net benefit. The curves (decision curves) indicate the net benefit of models (the reference model and 4 machine learning models) as well as 2 clinical alternatives (classifying no children as having the outcome vs classifying all children as having the outcome) over a specified range of threshold probabilities of outcome. Compared with the reference model, the net benefit for all machine learning models was greater across the range of threshold probabilities, except the net benefit for the random forest model was lower for threshold probabilities below approximately 3%.

Overall, 2352 children (4.5% of 52 037 visits) had the hospitalization outcome. The discrimination ability of different models, as represented by ROC curves, is shown in Figure 2A. The reference model had the lowest discriminative ability (C statistic, 0.73; 95% CI, 0.71-0.75) (Table 2), while all 4 machine learning models had a significantly higher discriminative ability (eg, C statistic for deep neural network, 0.80; 95% CI, 0.78-0.81; *P* < .001). Additionally, compared with the reference model, all machine learning models had a higher specificity (eg, 0.55 [95% CI, 0.52-0.58] in the reference model vs 0.75 [95% CI, 0.70-0.78] in the lasso regression [*P* < .001]) to predict the hospitalization outcome. With the relatively low prevalence of the hospitalization outcome (4%), the positive predictive value of all models was low (<0.15 for all) and the negative predictive value was high (>0.98 for all). While the reference model had the lowest positive likelihood ratio (1.82; 95% CI, 1.75-1.89), lasso regression achieved the highest positive likelihood ratio (2.71; 95% CI, 2.56-2.88). Specifically, the reference model identified all children who were hospitalized in triage levels 1 to 3 with a large number of overtriages (eTable 3 in the Supplement). By contrast, the machine learning models had a lower number of overtriaged children in triage levels 1 to 3 and correctly identified a larger number of hospitalized children in triage levels 4 and 5 (eTable 3 in the Supplement). In the decision curve analysis (Figure 2B), compared with the reference model, the net benefit for most machine learning models was greater across the range of threshold probabilities; the exception was the random forest model, which had a lower net benefit for thresholds below approximately 3%.

### Variable Importance

**Figure 3.**
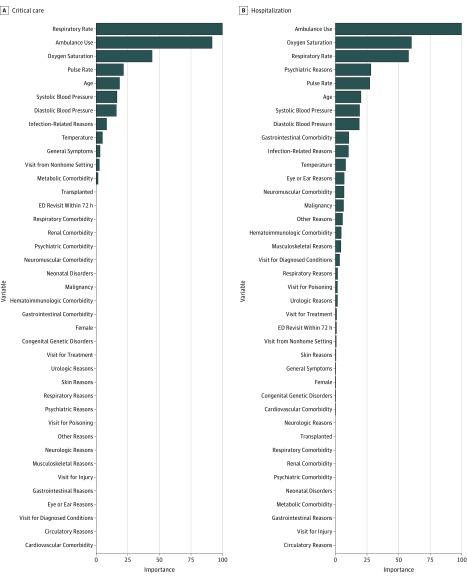
Importance of Each Predictor in the Gradient-Boosted Decision Tree Models The variable importance is a measure scaled to have a maximum value of 100. A, Critical care outcome. B, Hospitalization outcome. ED indicates emergency department.

Figure 3 demonstrates the variable importance in the gradient-boosted decision tree for each outcome. For both outcomes, age, vital signs (eg, oxygen saturation and respiratory rate), and arrival mode (ie, ambulance) were important predictors. The importance of these variables was consistent in the random forest models for each outcome (eFigure in the Supplement).

## Discussion

In this analysis of nationally representative data from 52 037 ED visits by children, we applied modern machine learning approaches (ie, lasso regression, random forest, gradient-boosted decision tree, and deep neural network) to ED triage classification and improved the overall discrimination ability to predict 2 clinical outcomes, critical care and hospitalization, as compared with the model using conventional triage approaches. The machine learning models achieved high predictive performance using only data routinely available at the time of triage (eg, visit reason, vital signs). These machine learning models also achieved a higher sensitivity for predicting the critical care outcome (ie, fewer undertriaged critically ill children) and a higher specificity for predicting the hospitalization outcome (ie, fewer overtriaged children who do not require inpatient management), while the reference model had a higher specificity for the critical care outcome and a higher sensitivity for the hospitalization outcome. Furthermore, the net benefit was also greater in the machine learning approaches across wide ranges of threshold probabilities (or clinical preferences to balance undertriage and overtriage). To our knowledge, this is the first study that has applied modern machine learning approaches specifically to a large ED population of children.

A key objective of ED triage is to promptly differentiate critically ill patients from the others and optimize ED resource allocation to both provide timely and high-quality care and also mitigate ED crowding and delayed care. However, the development of an accurate ED triage system for children remains challenging.^46,47^ The literature has demonstrated that the currently available triage systems (eg, pediatric emergency severity index) are subject to the clinician’s judgment^48^ and have suboptimal discrimination ability.^5,6,19,46,49^ Although adding a larger set of predictors (eg, detailed history of present illness, serial measurements of vital signs, physical examination) to a prediction model might improve the ability, it is not feasible at the ED triage setting owing to the limited information, resources, and time pressure. Alternatively, another strategy to assist clinicians in triage decision making is to leverage modern machine learning approaches that address complex nonlinear interrelations between predictors. Indeed, recent studies have reported that machine learning approaches improve predictions on traumatic brain injury in children,^10^ unplanned transfers to the ICU,^11^ in-hospital mortality in ED patients with sepsis,^12^ and hospitalization in patients with asthma or chronic obstructive pulmonary diseases.^13,14,15,50^ The present study builds on these prior reports, and extends them by demonstrating the superior ability of modern machine learning approaches to predict the clinical outcomes and disposition in a large sample of ED visits by children.

Emergency department triage systems strive for an appropriate balance between undertriage and overtriage because of the trade-offs between these 2 factors. In the current study, the machine learning approaches demonstrated a higher sensitivity for predicting the critical care outcome than the conventional approaches. Specifically, the machine learning approaches would reduce the number of undertriaged critically ill children in the conventional triage levels 3 to 5—children who received less attention in the ED. These findings lend support to the utility of these approaches at ED triage, in which one of the major priorities is to reduce undertriaging critically ill children. In contrast, as children who are going to be hospitalized do not necessarily require greater ED recourse (eg, children hospitalized for observation),^51,52^ the use of prediction models with a high sensitivity and low specificity for the hospitalization outcome may result in inefficient ED resource allocation, thereby further contributing to ED crowding and delay in care. Our machine learning approaches achieved a greater specificity and positive likelihood ratio for the hospitalization outcome, which would lead to fewer overtriages of children who might not require extensive resources in the ED. In particular, the machine learning approaches may reduce overtriage of children in triage levels 1 to 3 (immediate to urgent), for which greater resources are allocated. Moreover, in the decision curve analysis that accounts for the impact of false-negative (undertriage) and false-positive (overtriage), our machine learning approaches also demonstrated higher net benefit for both outcomes.

There are several potential explanations for the incremental gains in the prediction ability by the machine learning approaches. First, machine learning approaches are able to incorporate the high-order nonlinear interactions between predictors, which cannot be addressed by traditional modeling approaches (eg, logistic regression model).^16^ Additionally, we applied rigorous approaches to minimize potential overfitting of the models (eg, lasso and ridge regularization, cross-validation, and dropout). Moreover, the conventional ED triage systems for children rely on subjective and often variable evaluation of immediacy of medical need and projected resource use in the ED.^53,54^ Despite the superior prediction ability of machine learning compared with the conventional approaches, their prediction ability remained imperfect. The potential explanations include the subjectivity of data measurement (eg, visit reasons), contributions of clinical factors after ED triage (eg, timeliness and quality of ED management and response to treatment), differences in health behaviors of the patient and family, the clinician’s practice patterns, and institutional resources (eg, community hospital vs children’s hospital), or any combination of these factors. However, modern machine learning approaches possess scalability within a larger context of health information technology (eg, extracting a multitude of potential predictors from electronic health records and monitoring devices, continuous sophistication of the model using updated health data, and reinforcement learning).^55,56^ Indeed, the machine learning approaches have demonstrated potential to further improve their performance by integrating recently developed algorithms, such as natural language processing^57,58^ and diagnostically relevant facial gestalt information from images.^59^ Our observations and these recent developments collectively present reason for cautious optimism that machine learning approaches, as an assistive technology, further enhance the clinician’s triage decision making in a large ED population of children.

### Limitations

Our study has several potential limitations. First, we excluded visits with no information on the conventional triage classification, which might be a potential source of selection bias. Nevertheless, the patient characteristics and outcomes were comparable between the analytic and nonanalytic cohorts, arguing against significant bias. Second, the machine learning approaches are data driven and, therefore, depend on accurate data. While survey data might have some misclassification, the coding error rate was less than 1% in a 10% quality control sample of NHAMCS.^17^ Third, the imputation of missingness is a potential source of bias. However, the imputation by random forest is known to be a rigorous technique for imputation.^41^ Fourth, thresholds for the outcomes may be variable between the EDs (eg, different criteria for ICU admission). However, the decision curve analysis demonstrated greater net benefit for the machine learning approaches across the wide range of threshold probabilities (or clinical preferences). Additionally, these approaches are highly flexible and inherently adaptive to local care systems, distinguishing them from the conventional triage systems. Fifth, one may surmise that the small number of outcomes might have affected the prediction ability. But all 4 machine learning approaches consistently demonstrated superior predictive ability compared with the conventional approach. Sixth, NHAMCS data do not measure some clinical variables (eg, patient appearance, clinician’s gestalt, medications, and prehospital treatment and response). However, the objective of the present study was not to derive prediction models using a broad set of predictors but to develop machine learning models to harness a limited set of clinical data that are currently available in the typical ED triage setting.

## Conclusions

In this analysis of nationally representative data of children presenting to the ED, by using data routinely available at the time of triage, we found that the application of machine learning approaches to ED triage improved the discriminative ability to predict clinical and disposition outcomes compared with the conventional triage approach. Additionally, the machine learning approaches achieved a high sensitivity for predicting the critical care outcome. Specifically, these approaches would reduce the number of undertriaged critically ill children in the conventional triage levels 3 to 5 (ie, children who would be missed by conventional approaches). Additionally, while conventional approaches may help clinicians better identify children who require hospitalization, the machine learning approaches had a higher specificity for predicting the hospitalization outcome, which would avoid overtriaging children who are less ill and may not require extensive ED resources. Although external prospective validation is needed, our findings present an opportunity to apply advanced prediction approaches to support the clinician’s ED triage decision making, which may, in turn, achieve more accurate clinical care and optimal resource allocation.

## Notes

Supplement. **eTable 1.** Missingness in Predictors in Emergency Department Children (n=52,037) **eTable 2.** Predictor Variables and Outcomes Between Analytic and Non-analytic Cohort **eTable 3.** The Number of Actual Outcomes and Predicted Outcomes of Prediction Models in the Test Set **eFigure.** Importance of Each Predictor in the Random Forest Models
