# Supplementary material for: Machine Learning–Based Prediction of Clinical Outcomes for Children During Emergency Department Triage
Source: JAMA Netw Open. 2019 Jan 11;2(1):e186937. doi: 10.1001/jamanetworkopen.2018.6937 (PMC6484561; doi:10.1001/jamanetworkopen.2018.6937)
Supplement: Supplement. — eTable 1. Missingness in Predictors in Emergency Department Children (n=52,037) eTable 2. Predictor Variables and Outcomes Between Analytic and Non-analytic Cohort eTable 3. The Number of Actual Outcomes and Predicted Outcomes of Prediction Models in the Test Set eFigure. Importance of Each Predictor in the Random Forest Models [file jamanetwopen-2-e186937-s001.pdf]

Supplementary Online Content

Goto T, Camargo CA Jr, Faridi MK, Freishtat RJ, Hasegawa K. Machine learning–based prediction of clinical outcomes for children during emergency department triage. *JAMA Netw Open*. 2019;2(1):e186937. doi:10.1001/jamanetworkopen.2018.6937

eTable 1. Missingness in Predictors in Emergency Department Children (n=52,037)
eTable 2. Predictor Variables and Outcomes Between Analytic and Non-analytic Cohort
eTable 3. The Number of Actual Outcomes and Predicted Outcomes of Prediction Models in the Test Set
eFigure. Importance of Each Predictor in the Random Forest Models

This supplementary material has been provided by the authors to give readers additional information about their work.

© 2019 Goto T et al. *JAMA Network Open.*

eTable 1. Missingness in predictors in emergency department children (n=52,037)

Variables	Missing, n (%)
Age	0 (0)
Sex	0 (0)
Mode of arrival	2,143 (4)
Vital signs	
Temperature	1,809 (3)
Pulse rate	4,936 (8)
Systolic blood pressure	19,701 (38)*
Diastolic blood pressure	20,036 (38)*
Respiratory rate	2,854 (5)
Oxygen saturation	9,650 (19)
Chief complaints	115 (0.2)

*81% of children with missing data were aged ≤5 years

© 2019 Goto T et al. *JAMA Network Open.*

eTable 2. Predictor variables and outcomes between analytic and non-analytic cohort

	Analytic cohort	Non-analytic cohort
**Variables**	n=52,037	n=10,792
Age (year), median (IQR)	6 (2-14)	6 (2-13)
Female sex	24,929 (48)	5,232 (48)
Mode of arrival		
Ambulance	3,637 (7)	586 (5)
Vital signs		
Temperature (F), median (IQR)	98.4 (97.9-99.2)	98.2 (97.5-99.0)
Pulse rate (bpm), median (IQR)	105 (87-128)	112 (89-150)
Systolic blood pressure (mmHg), median (IQR)	109 (104-120)	115 (105-126)
Diastolic blood pressure (mmHg), median (IQR)	62 (66-72)	69 (61-76)
Respiratory rate (per min), median (IQR)	20 (18-24)	22 (18-30)
Oxygen saturation (%), median (IQR)	99 (98-100)	99 (98-100)
Major chief complaints		
General (e.g. fever) complaints	9,161 (18)	1,803 (17)
Respiratory-related complaints	8,201 (16)	1,792 (17)
Gastrointestinal-related complaints	6,677 (13)	1,405 (13)
Musculoskeletal-related complaints	4,489 (9)	835 (8)
Eye and ear-related complaints	2,912 (6)	658 (6)
Injuries	10,594 (20)	2,073 (19)
ED visit from patient's home	51,584 (99)	10,280 (95)
ED revisit within 72 hours	1,390 (3)	352 (3)
Pediatric complex chronic condition (≥1)	291 (1)	67 (1)
Outcomes		
Critical care outcome*	163 (0.3)	8 (0.1)
Hospitalization outcome†	2,352 (4)	315 (3)

Data were presented as unweighted number (percentage) of patients unless otherwise indicated
Abbreviations: ED, emergency department; IQR, interquartile range
* Direct admission to intensive care unit (ICU) or in-hospital death
† Admission to an inpatient care site or direct transfer to an acute care hospital

© 2019 Goto T et al. *JAMA Network Open.*

eTable 3. The number of actual outcomes and predicted outcomes of prediction models in the test set

		Reference model*		Lasso regression		Random forest		Gradient boosted tree		Deep neural network	
**Conventional triage category**	**Number of ICU admissions or in-hospital deaths, n (%)**	**Number of correctly identified outcomes**	**Number of predicted outcomes**	**Number of correctly identified outcomes**	**Number of predicted outcomes**	**Number of correctly identified outcomes**	**Number of predicted outcomes**	**Number of correctly identified outcomes**	**Number of predicted outcomes**	**Number of correctly identified outcomes**	**Number of predicted outcomes**
1: Immediate (n=255)	6 (2.4%)	6	255	6	79	6	102	6	104	5	76
2: Emergent (n=1227)	16 (1.3%)	16	1227	13	372	14	489	14	451	14	336
3: Urgent (n=5829)	12 (0.2%)	0	0	5	1003	8	1410	7	1339	8	1051
4: Semi-urgent (n=6597)	4 (0.1%)	0	0	3	660	3	1049	3	965	3	734
5: Non-urgent (n=1703)	3 (0.2%)	0	0	1	134	2	249	2	204	2	151
Overall (n=15611)	41 (0.3%)	22	1482	28	2248	33	3299	32	3063	32	2348
		**Reference model***		**Lasso regression**		**Random forest**		**Gradient boosted tree**		**Deep neural network**	
**Conventional triage category**	**Number of hospitalization outcome, n (%)**	**Number of correctly identified outcomes**	**Number of predicted outcomes**	**Number of correctly identified outcomes**	**Number of predicted outcomes**	**Number of correctly identified outcomes**	**Number of predicted outcomes**	**Number of correctly identified outcomes**	**Number of predicted outcomes**	**Number of correctly identified outcomes**	**Number of predicted outcomes**
1: Immediate (n=255)	50 (19.6%)	50	255	42	112	33	87	37	110	33	85
2: Emergent (n=1227)	183 (14.9%)	183	1227	141	592	118	410	121	489	102	372
3: Urgent (n=5829)	335 (5.8%)	335	5829	219	1940	156	1153	171	1518	155	1195
4: Semi-urgent (n=6597)	104 (1.6%)	0	0	50	1246	33	766	47	1187	39	831
5: Non-urgent (n=1703)	20 (1.2%)	0	0	15	289	6	181	9	269	8	178
Overall (n=15611)	692 (4.4%)	568	7311	467	4179	346	2597	385	3573	337	2661

Abbreviation: ICU, intensive care unit
To address the low proportion of outcome, the threshold of prospective prediction results were based on receiver operating characteristic curve (i.e., the value with the shortest distance to the perfect model)
* Logistic regression model using the conventional triage classification (categories 1 [immediate] to 5 [non-urgent]).

© 2019 Goto T et al. *JAMA Network Open.*

Importance of Each Predictor in the Random Forest Models
eFigure A

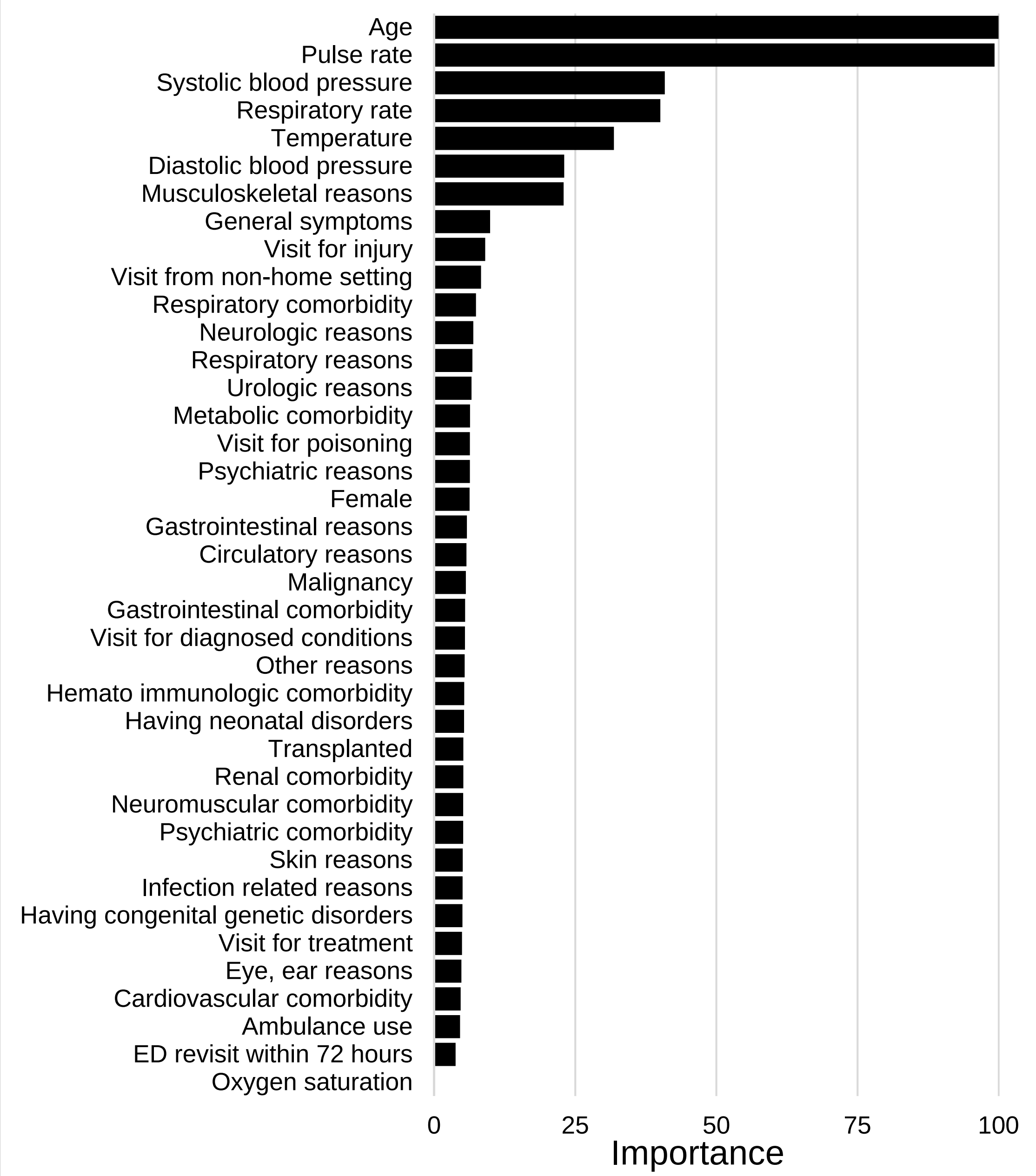

© 2019 Goto T et al. *JAMA Network Open.*

eFigure B

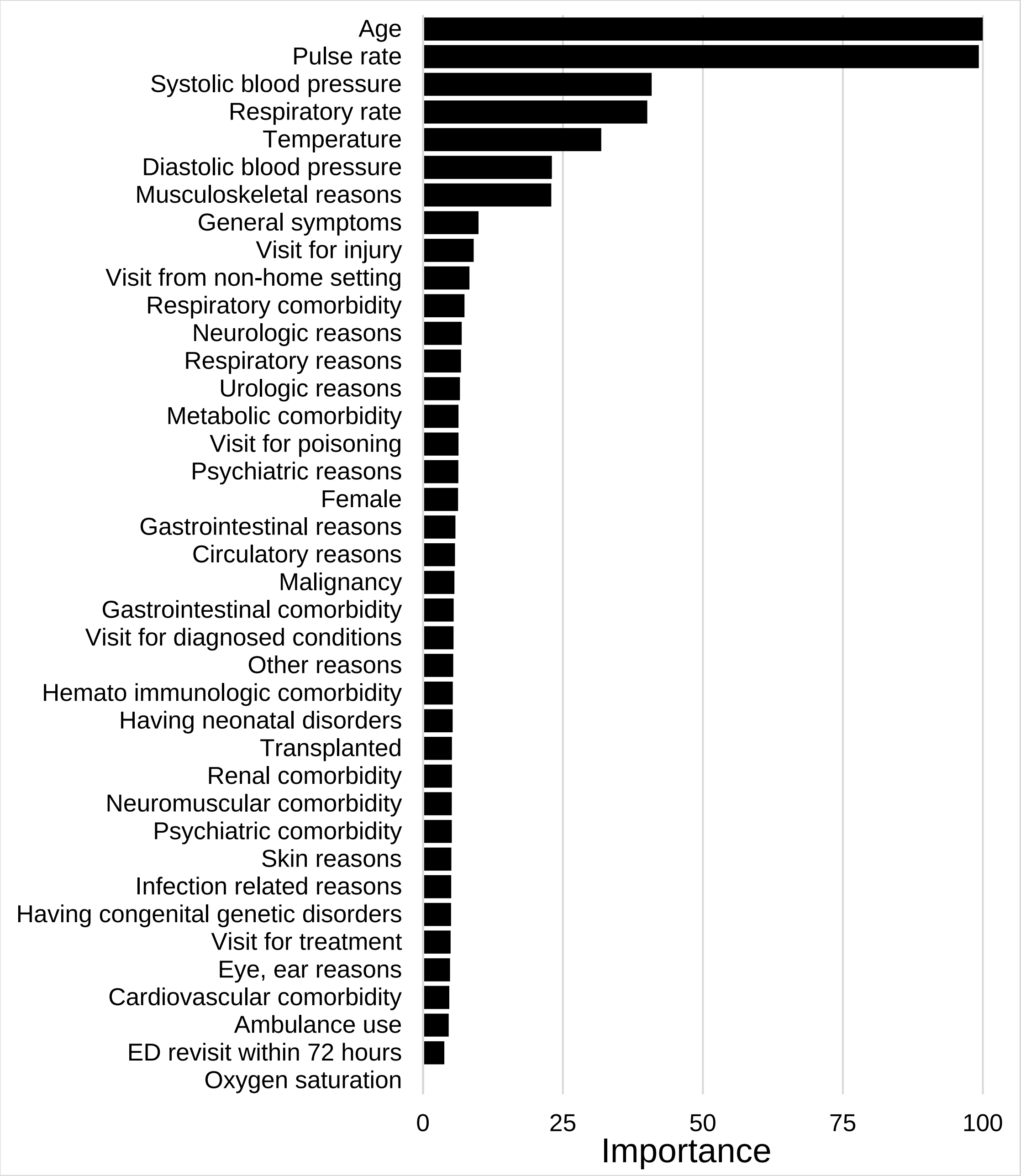

© 2019 Goto T et al. *JAMA Network Open.*
